# Supplementary material for: Reconciling Mining with the Conservation of Cave Biodiversity: A Quantitative Baseline to Help Establish Conservation Priorities
Source: PLoS One. 2016 Dec 20;11(12):e0168348. doi: 10.1371/journal.pone.0168348 (PMC5173368; doi:10.1371/journal.pone.0168348)
Supplement: S1 Dataset — (ZIP) [file pone.0168348.s002.zip › Taxa/Serra Norte/SN_2013/N5S/Lista N5S-01.pdf]

CAVIDADE N5S-0001

Classe	Ordem	Fam/Outros	Gên/Outros	Espécie	S-U	U-S	Zon.
Arachnida	Acari	Eupodidae		sp.1	X		P
Arachnida	Acari	Rhagidiidae		sp.20	X		P
Arachnida	Amblypygi	Charinidae	*Charinus*	sp.1	2		P
Arachnida	Amblypygi	Phrynidae	*Heterophrynus*	*longicornis*	3	X	P
Arachnida	Araneae	Ochyroceratidae	*Gen*.n.	sp.1		X	
Arachnida	Araneae	Ochyroceratidae	*Ochyrocera*	sp.		X	
Arachnida	Araneae	Pholcidae	*Mesabolivar*	sp.1	X	X	P
Arachnida	Araneae	Scytodidae	*Scytodes*	sp.1	6		P
Arachnida	Araneae	Theridiidae	*Theridion*	sp.1	X		P
Arachnida	Araneae	Theridiosomatidae	*Plato*	sp.1	X	X	P
Arachnida	Opiliones	Cosmetidae	*Roquettea*	*singularis*	1	X	P
Chilopoda	Scutigeromorpha	Pselliodidae	*Sphendononema*	*guildingii*	1	X	
Diplopoda	Polydesmida	Chelodesmidae		sp.3	3		P
Entognatha	Collembola	Paronellidae		sp.1	X		P
Entognatha	Diplura	Campodeidae		sp.1	X	X	P
Insecta	Blattodea			jovem	1		P
Insecta	Coleoptera	Carabidae		sp.3	X		P
Insecta	Coleoptera	Carabidae		sp.13	X		P
Insecta	Diptera	Cecidomyiidae	Cecidomyiinae	sp.	X		P
Insecta	Diptera	Culicidae	*Culex*	sp.	X		P
Insecta	Diptera	Psychodidae	Phlebotominae	sp.	X		P
Insecta	Homoptera	Cixiidae		jovem	X	X	P
Insecta	Hymenoptera	Formicidae	*Brachymyrmex*	sp.1	X		P
Insecta	Hymenoptera	Formicidae	*Camponotus*	sp.2	X		P
Insecta	Hymenoptera	Formicidae	*Dolichoderus*	aff. *bispinosus*	X		P
Insecta	Hymenoptera	Formicidae	*Gnamptogenys*	sp.1	X		P
Insecta	Hymenoptera	Formicidae	*Hypoponera*	sp.		X	
Insecta	Hymenoptera	Formicidae	*Pachycondyla*	sp.1	X		P
Insecta	Hymenoptera	Formicidae	*Pheidole*	sp.		X	
Insecta	Hymenoptera	Formicidae	*Pseudomyrmex*	aff. *opaciceps*	X		P
Insecta	Lepidoptera	Noctuoidea		sp.3	1		P
Insecta	Orthoptera	Phalangopsidae	*Paraclodes*	sp.	10	X	P
Insecta	Orthoptera	Phalangopsidae	*Phalangopsis*	sp.		X	P
Insecta	Orthoptera	Tettigoniidae	Listroscelidinae	sp.	1		P
Insecta	Psocoptera			jovem		X	
Gastropoda	Pulmonata	Systrophiidae	*Happia*	sp.		X	
Malacostraca	Isopoda	Philosciidae		sp.		X	
Symphyla		Scutigerellidae		sp.	X	X	P
Amphibia	Anura	Strabomantidae	*Pristimantis*	cf. *fenestratus*		1	P
Mammalia	Chiroptera	Emballonuridae	*Peropteryx*	sp.		X	
